# Acoustic Insulation Mechanism of Membrane-Type Acoustic Metamaterials Loaded with Arbitrarily Shaped Mass Blocks of Variable Surface Density

**DOI:** 10.3390/ma15041556

**Published:** 2022-02-18

**Authors:** Junyu Li, Yuanyuan Shi, Renjie Jiang, Zhifu Zhang, Qibai Huang

**Affiliations:** 1 State Key Laboratory of Digital Manufacturing Equipment and Technology, School of Mechanical Science and Engineering, Huazhong University of Science and Technology, Wuhan 430074, China; lmagikarp@hust.edu.cn (J.L.); D201980200@hust.edu.cn (Y.S.); jrj6320@163.com (R.J.); jeff.zfzhang@foxmail.com (Z.Z.); 2 Hubei Institute of Specialty Vehicle, Suizhou 441300, China

**Keywords:** membrane-type acoustic metamaterials, sound transmission loss, variable surface density, conformal mapping

## Abstract

Membrane-type acoustic metamaterials (MAMs) have recently received widespread attention due to their good low-frequency sound-transmission-loss (STL) performance. A fast prediction method for the STL of rectangular membranes loaded with masses of arbitrary shapes and surface density values is proposed as a semi-analytical model for calculating the STL of membrane-type acoustic metamaterials. Through conformal mapping theory, the mass blocks of arbitrary shapes were transformed into regular shapes, which simplified the calculation model of acoustic propagation loss prediction, and the prediction results were verified by finite element simulations. The results show that the change in mass surface density was closely related to the size and frequency distribution of STL. The influence of the mass center on the STL and characteristic frequency of the film metamaterial is discussed.

## 1. Introduction

In order to reduce noise, many new material structures and technologies have been developed, such as sound barriers [1] and porous materials [2], which have a wide range of applications in the aviation industry, the automotive industry and the appliance industry. However, these technical means are often ineffective in the low-frequency band. As a result, acoustic metamaterials have emerged for solving low-frequency noise problems.

Thin-film acoustic metamaterials have the advantages of being light, thin and ductile and are of great interest because they can guarantee good low-frequency sound insulation while meeting the needs of lightweight, non-planar structures. Research on thin-film acoustic metamaterials has focused on two aspects, (1) structural design and (2) theoretical study of sound transmission loss (STL).

In terms of structural design, Yang et al. [3] fabricated an acoustic metamaterial with broadband double-negative characteristics using paired thin-film structures. Zhang et al. [4] used a circular local resonant film structure model for vibration and noise reduction design and analyzed the factors influencing band gap width and STL characteristics. William et al. [5] prepared a locally resonant membrane-type acoustic metamaterial array loaded with toroidal masses and analyzed the relationship between a single cell and multiple MAMs arrays. Guancong Ma et al. [6] established a membrane-type metamaterial structure with cavities to increase the noise reduction effect of the structure by using hybrid resonance. Huang et al. [7] proposed a petal-shaped circular membrane-type metamaterial that is lighter than the cylindrical structure while ensuring certain STL effect. Lu et al. [8] investigated the STL in the eccentric state of the mass block and introduced a preparation technique to accurately apply tension to the film. Zhou et al. [9] relied on experimental methods to investigate the effect of different forms of mass blocks on the STL of membrane-type metamaterials in the low-frequency band and found that the reasonable combination of multiple mass blocks of different shapes could effectively broaden the sound attenuation region in the low frequency domain. Li et al. [10] proposed a membrane-type metamaterial with a honeycomb structure and found, through experiments, that changing the size of a single cell could adjust its STL. Gao et al. [11] designed a double-layer thin-film metamaterial and analyzed its bandgap characteristics, demonstrating that the frequency band generated by the bandgap could be changed by adjusting the film tension and mass block quality; later, Cai et al. [12] investigated the low-frequency STL performance of asymmetrically coupled thin-film acoustic metamaterials by the finite element method and found that the STL performance could be tuned by changing the position and distribution of the mass blocks.

However, for these structures, the technical means of experiments and simulations are often used to analyze their STL and studies of the STL theoretical aspects of membrane-type metamaterials are lacking.

As a result, researchers have begun to investigate the STL theoretical aspects from membrane-type metamaterials. Firstly, Kornhauser and Mintzer [13] and Cohen and Handelman [14] analyzed the STL effect of a circular mass block when it is concentric with a circular film by studying the intrinsic frequency and vibration pattern of the circular film. Zhang et al. [15] proposed an analytical method in which the solid mass is located in the center of the film through the Galerkin method. Later, Tian et al. [16] derived an analytical model based on Zhang’s analytical method when the circular ring is located at the center of the circular film. Considering the stiffness and inertia of the mass block, Chen [17] et al. proposed a point-matching method to calculate membrane-type metamaterials through analytical methods, but this method needs to solve a nonlinear eigenvalue problem, which requires a large amount of calculation. Langfeldt et al. [18,19] introduced a method based on the grid convergence index to accelerate the calculation speed of the point-matching method on the basis of Chen. However, for researchers facing different MAMs structures, there is a big challenge in terms of calculating the eigenmode of a different film and the mathematical solution of the mass inertia and stiffness.

This paper puts forward a semi-analytical model for STL fast prediction of arbitrary shape area density structures, which effectively simplifies the calculation difficulty of complex mass film metamaterials [18]. This model extends the limit of the literature [15] that can only calculate the regular mass block.

To this end, an analytical model of STL under a regular mass block with a fixed film load at the perimeter was first derived. On this basis, the theory of conformal mapping of irregular mass blocks was introduced and the surface density of irregular mass blocks was obtained by finite element techniques to establish a semi-analytical model for the surface density of arbitrary shapes. Then, the semi-analytical method proposed in this paper was verified by relying on the theoretical model and the finite element model in Ref. [18]. Finally, the effect of objective factors of the mass block on the acoustic transmission loss of membrane-type metamaterials is discussed.

## 2. Analytical Model and Formulation

In this section, starting from a common rectangular mass block, based on the calculation formula in Ref. [15], the vibration equations and mode oscillations of membrane-type metamaterials under fixed constraints were derived and the STL of thin films with additional rectangular mass blocks were solved based on the modal superposition method and Rayleigh’s method. The conformal mapping theory was extended to calculate the STL model for rectangular films with arbitrarily shaped mass blocks. Finally, relying on finite element techniques, a semi-analytical method for solving membrane-type acoustic metamaterials with arbitrarily shaped and variable surface density mass blocks was developed for calculating STL.

### 2.1. Loaded Mass Blocks of Any Shape

From the work of William T. Edwards et al. [5], it can be found that different shapes of mass blocks can produce different STL effects on a metamaterial cell element. However, there are no general analytical methods for calculating the effects of differently shaped mass blocks. In this regard, the conformal mapping theory is introduced to convert any shape mass block into a regular rectangular mass block.

**Figure 1:**
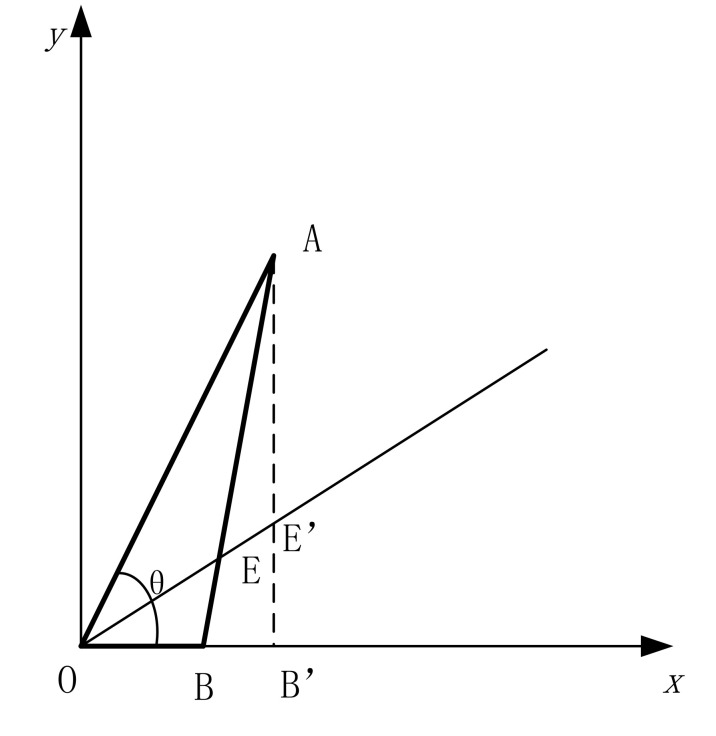
Mapping of the segmented irregular triangular area.

The area occupied by the contact surface of the arbitrarily shaped mass block and the film is mapped into a rectangular area using the analytical mapping technique; a coordinate axis is established at the center of the arbitrarily shaped figure and the arbitrarily shaped figure is divided into an infinite number of angle-determined triangles using the coordinate origin as the top angle, then an angle-preserving mapping is performed for each triangle to finally obtain a mapping function for the whole figure. The mapping of the partitioned triangle is shown in Figure 1: make an arbitrary line intersecting *AB* and *AB’* and set the points *E* and *E’*, with the coordinates of point *A* being (a, a*tan*θ*) and the coordinates of point *B* being (*b*, 0); and map points *E* to *E’* to obtain the mapping Equation (1).

(1) $$L_{\left(x,y\right)}=\left[\begin{array}{c} \frac{a\tan\theta -k\left(a-b\right)}{ab\tan\theta }x\\ \frac{a\tan\theta -k\left(a-b\right)}{ab\tan\theta }y \end{array}\right]$$

where $k=\frac{y}{x}$.

The mapping function is obtained after the transformation into a rectangle; then, the coordinates are transformed so that the origin of the established coordinate system corresponds to the center of mass of the mass block on the membrane-type metamaterial. As shown in Equation (2),

(2) $$H\left(x,y\right)=L_{\left(x,y\right)}+\left[\begin{array}{c} x_{n}\\ y_{n} \end{array}\right]$$

where *x_n_*, *y_n_* is the distance of the coordinate transformation.

**Figure 2:**
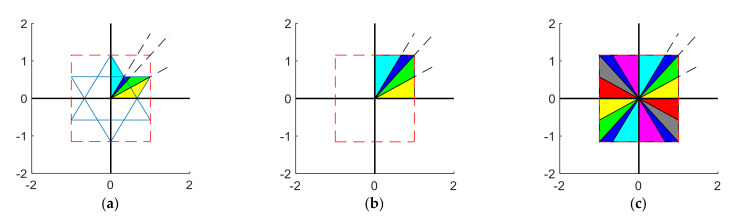
Mapping of hexagons into several triangles: (**a**) 1/4 hexagonal division method; (**b**) 1/4 hexagonal mapping into small rectangles; (**c**) hexagon divided into 16 small triangles for mapping.

Taking a hexagon consisting of two square triangles with sides of 2 mm, as shown in Figure 2, the conformal mapping can be expressed as

(3) $$L_{1}\left(\left[{}_{y}^{x}\right]\right)=\left[{}_{\frac{3-\sqrt{3}\frac{y}{x}}{2}y}^{\frac{3x}{2}-\sqrt{3}\frac{y}{2}}\right],L_{2}\left(\left[{}_{y}^{x}\right]\right)=\left[{}_{\sqrt{3}\frac{y^{2}}{x}}^{\sqrt{3}y}\right],L_{3}\left(\left[{}_{y}^{x}\right]\right)=\left[{}_{2y}^{2x}\right],L_{4}\left(\left[{}_{y}^{x}\right]\right)=\left[{}_{\left(\frac{\sqrt{3}x+y}{y}\right)y}^{\left(\frac{\sqrt{3}x+y}{y}\right)x}\right]\;L_{5}\left(\left[{}_{y}^{x}\right]\right)=\left[{}_{\left(\frac{y-\sqrt{3}x}{y}\right)y}^{\left(\frac{y-\sqrt{3}x}{y}\right)x}\right],L_{6}\left(\left[{}_{y}^{x}\right]\right)=\left[{}_{2y}^{2x}\right],L_{7}\left(\left[{}_{y}^{x}\right]\right)=\left[{}_{-\sqrt{3}\frac{y^{2}}{x}}^{\sqrt{3}x}\right],L_{8}\left(\left[{}_{y}^{x}\right]\right)=\left[{}_{\left(\frac{3+\sqrt{3}\frac{y}{x}}{2}\right)y}^{\left(\frac{3+\sqrt{3}\frac{y}{x}}{2}\right)x}\right]$$

The hexagon is an axisymmetric figure, so the mapping function of *L*_9-16_, which is the same as *L*_1-8_, is not repeated.

The mapping function for a rectangular membrane-type metamaterial with a side length of 20 mm located at its center is shown in Equation (4).

(4) $$H_{i\left(x,y\right)}=\left[\begin{array}{c} 1\\ \frac{\sqrt{3}}{2} \end{array}\right]L_{i\left(x,y\right)}+\left[\begin{array}{c} 10\\ 10 \end{array}\right]$$

where $\left[\begin{array}{c} 1\\ \frac{\sqrt{3}}{2} \end{array}\right]$ denotes the scaling of the rectangle to a square; *i* = 1, 2, 3, …, 16.

In the above-mentioned way, different shapes of mass blocks can be transformed into rectangular mass blocks.

### 2.2. STL Theory of Membrane-Type Acoustic Metamaterials

After a film has been disturbed by an external force, the force that restores it to equilibrium is mainly tension, while the elastic stiffness of the material itself is negligible compared to tension. Therefore, the influence of the elastic stiffness of the film material itself on the film vibration is not considered in the vibration analysis of the film.

**Figure 3:**
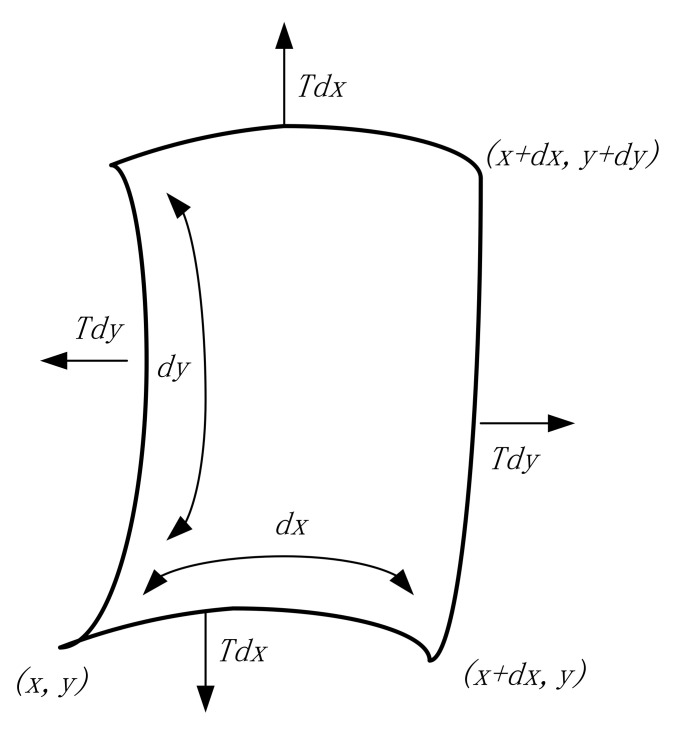
Forces during transverse vibration of the unitary membrane.

Let us suppose that a rectangular film in the xy-plane vibrates transversely with small amplitude and is uniformly stretched in all directions, subject to a tension *T* per unit length in N/m. The mass per unit area of the film is $\rho_{s}$, called the surface density, and a unit surface dxdy is taken on the film; when the film is deformed, it is subjected to a tension in kg/m^3^ at its edges by the adjacent elementary segment, as shown in Figure 3. Let the angle of tension *T* with respect to the x coordinate be α. In the case of small amplitudes, the vertical component of tension acting on the x end of this unit film can be regarded as Ttanα. In the Cartesian coordinate system, *w*(*x*, *y*, *t*) represents the transverse displacement of a point (*x*, *y*) on the membrane at moment t. Then, we have $\tan\alpha ={\left(\frac{\partial w}{\partial x}\right)}_{x}$; thus, the force acting vertically on the entire *dy* edge at the x end is $T{\left(\frac{\partial w}{\partial x}\right)}_{x}dy$ and the force in the vertical direction on the entire edge of *dy* at the *x* + *dx* end should be $T{\left(\frac{\partial w}{\partial x}\right)}_{x+dx}dy$. This gives the combined force in the vertical direction on the edge of this face element *x* and *x + dx*, as shown in Equation (5).

(5) $$T{\left(\frac{\partial w}{\partial x}\right)}_{x+dx}dy-T\left(\frac{\partial w}{\partial x}\right)dy=T\left(\frac{\partial^{2}w}{\partial x^{2}}\right)dxdy$$

Similarly, the combined force in the vertical direction on the edge of *y* and *y + dy* is

(6) $$T{\left(\frac{\partial w}{\partial y}\right)}_{y+dy}dx-T\left(\frac{\partial w}{\partial y}\right)dx=T\left(\frac{\partial^{2}w}{\partial y^{2}}\right)dxdy$$

The equation of motion of the rectangular membrane is obtained from Newton’s second law, as follows:

(7) $$T\left(\frac{\partial^{2}w}{\partial x^{2}}+\frac{\partial^{2}w}{\partial y^{2}}\right)dxdy=\rho_{s}dxdy\left(\frac{\partial^{2}w}{\partial t^{2}}\right)$$

After rectification, the vibration equation of the rectangular film [20] is

(8) $$T\left(\frac{\partial^{2}w}{\partial x^{2}}+\frac{\partial^{2}w}{\partial y^{2}}\right)=\rho_{s}\left(\frac{\partial^{2}w}{\partial t^{2}}\right)$$

The equation for the free vibration of a rectangular film can be written as

(9) $$\rho_{s}\left(\frac{\partial^{2}w}{\partial t^{2}}\right)=T\nabla^{2}w$$

where $\nabla^{2}=\frac{\partial^{2}}{\partial x^{2}}+\frac{\partial^{2}}{\partial y^{2}}$ is the Laplace operator in a two-dimensional coordinate system.

**Figure 4:**
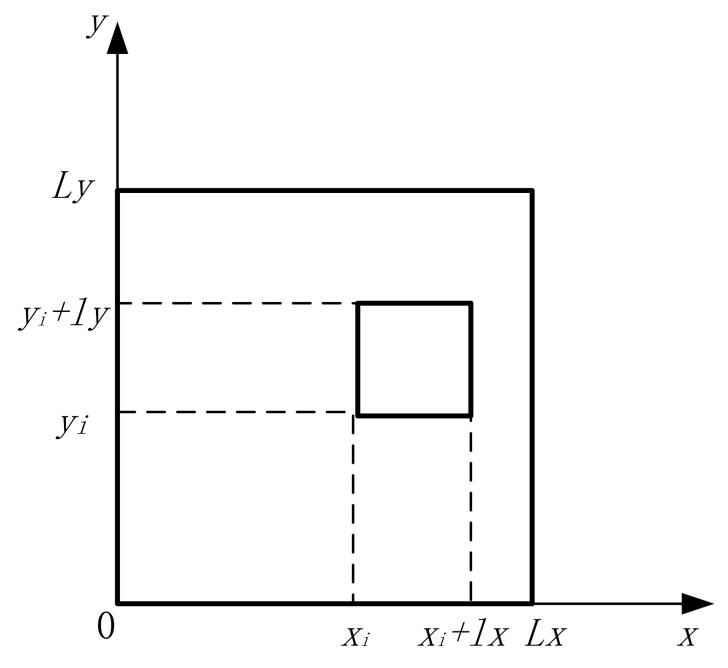
Additional distributed mass on the film surface at fixed constraints.

Fix the four sides of the rectangular film and glue a mass of a certain size and weight to the film (see Figure 4). It is assumed that the additional mass does not affect the deformation of the film at the position of the mass block, i.e., the effect of the bending stiffness of the mass block on the transverse vibration of the film is neglected, the width of the *i*th mass block on the film is *lx_i_*, the length is *ly_i_*, the surface density is denoted by *ρi* and the point (*xi*, *yi*) denotes the coordinates of the nearest corner of the *i*th mass block from the coordinate origin. The free vibration equation for the added mass membrane structure is obtained by treating the inertial force generated by the added mass as an applied excitation force acting on the vibration equation of the membrane.

(10) $$\rho_{s}\frac{\partial^{2}w}{\partial t^{2}}+\sum_{i=1}^{I}\rho_{i}\hbar \left(x,y,x_{i},y_{i},l_{xi},l_{yi}\right)\frac{\partial^{2}w}{\partial t^{2}}-T\nabla^{2}w=0$$

Solving this, Equation (11), based on modal superposition theory, yields the transverse vibration displacement of a rectangular film [21].

(11) $$w(x,y,t)=\sum_{n=1}^{N_{x}}\sum_{m=1}^{N_{y}}W_{nm}(x,y)q_{nm}(t)$$

where $w_{nm}\left(x,y\right)$ is the modal function of each order for a uniform membrane structure without concentrated mass.

For a rectangular membrane structure with fixed boundaries,

(12) $$W_{nm}(x,y)=\sin\frac{n\pi }{L_{x}}x\sin\frac{m\pi }{L_{y}}y$$

Under simple harmonic excitation, $q_{nm}(t)={\tilde{q}}_{nm}e^{j\omega t}$. Substituting Equation (11) into Equation (10), multiplying both sides of the equation collectively by *Wrs(x,y)* and integrating over the entire surface of the membrane structure (0 ≤ *x* ≤ *L_x_*, 0 ≤ *y* ≤ *L_y_*), Equation (13) can be obtained.

(13) $$-\omega^{2}\rho_{s}M_{rs}{\tilde{q}}_{rs}-\omega^{2}\sum_{i=1}^{I}\rho_{i}\sum_{n=1}^{N_{x}}\sum_{m=1}^{N_{y}}I_{nm,rs}^{i}{\tilde{q}}_{nm}+TK_{rs}{\tilde{q}}_{rs}=0\;r=1,2,\ldots ,N_{x},s=1,2,\ldots ,N_{y}$$

where

(14) $$M_{rs}=\int_{0}^{L_{x}}\int_{0}^{L_{y}}W_{r3}\sum_{n=1}^{N_{x}}\sum_{m=1}^{N_{y}}W_{nm}dxdy$$

(15) $$K_{rs}=-\int_{0}^{L_{x}}\int_{0}^{L_{y}}W_{r3}\nabla^{2}\sum_{n=1}^{N_{x}}\sum_{m=1}^{N_{y}}W_{xm}dxdy$$

(16) $$I_{nm,rs}^{i}=\int_{x}^{x_{i}+l_{n}}\int_{y}^{y_{y}+l_{n}}W_{rs}W_{nm}dxdy$$

Considering a train of planar acoustic waves of frequency ω incident perpendicular to the surface of the film from the negative direction of the z-axis, Equation (13) can be rewritten as

(17) $$-\omega^{2}\rho_{s}M_{mn}{\tilde{q}}_{n}-\omega^{2}\rho_{m}\sum_{n}^{N}I_{m,n}{\tilde{q}}_{n}+2j\omega \rho_{a}c_{a}C_{mn}{\tilde{q}}_{n}+TK_{mn}{\tilde{q}}_{n}=2\tilde{A}H_{m}$$

where $\tilde{A}$ is the incident sound pressure amplitude.

Others,

(18) $$M_{mn}=\int_{0}^{L_{t}}\int_{0}^{L_{y}}W_{m}\sum_{n}^{N}W_{n}dxdy$$

(19) $$I_{m,n}=\int_{x_{0}}^{x_{0}+l_{\mathrm{xi}}}\int_{y_{0}}^{y_{0}+l_{\mathrm{yi}}}W_{m}W_{n}dxdy$$

(20) $$K_{mn}=-\int_{0}^{L_{t}}\int_{0}^{L_{y}}W_{m}\nabla^{2}\sum_{n}^{N}W_{n}dxdy$$

(21) $$C_{mn}=\int_{0}^{L_{r}}\int_{0}^{L_{y}}W_{m}\sum_{n}^{N}W_{n}dxdy,$$

(22) $$H_{m}=\int_{0}^{L_{t}}\int_{0}^{L,}W_{m}dxdy$$

Considering the irregular shape of the mass, substitute Equation (4) into Equation (19) to obtain Equation (23).

(23) $$I_{m,n}=\int_{x_{0}}^{x_{0}+l_{\mathrm{xi}}}\int_{y_{0}}^{y_{n}+l_{\mathrm{yi}}}W_{m}W_{n}H_{\left(x,y\right)}dxdy$$

In this way, the effect of loading any shape mass on MAMs can be reflected.

Solving for the rectangular film STL by Rayleigh’s method and writing it in matrix form gives Equation (24).

(24) $$t_{p}=\frac{\langle \tilde{C}\rangle }{\tilde{A}}=\frac{2\rho_{1}c_{1}\omega }{L_{x}L_{y}}{\left\{H\right\}}^{T}\frac{1}{j\omega^{2}\{[M]+[Q]\}+\omega [C]+j[K]}\{H\}$$

where [Q] = $\rho_{m}\left[l\right]$,$\rho_{m}$ is the surface density of the mass block.

This leads to Equation (25) for the STL of membrane-type acoustic metamaterials.

(25) $$TL=20\log_{10}\left(1/t_{p}\right)$$

### 2.3. Loading Mass Blocks with Any Area Density 

In the analysis of membrane-type metamaterials, the influence of the elastic stiffness of the mass block itself is ignored and its influence on STL is characterized by its surface density. The mass of arbitrary structural shapes is loaded on the film, which also leads to inconsistent areal density of MAMs. The area density of mass blocks with arbitrary material composition and height can be obtained using finite element simulation software (COMSOL 5.6, Stockholm, Sweden). The area density function *rho* (*x*, *y*) can be obtained by interpolation and substituted into Equation (24), so as to calculate the STL of mass blocks with arbitrary area density.

## 3. Validation of the Method and Discussions

Firstly, this section compares the STL results of membrane-type metamaterial models in the literature [18] to verify the reliability of the semi-analytical theory in this paper. Then, the finite element analysis model of rectangular membrane-type metamaterials, established by using multiphysics field analysis software COMSOL 5.6, and a metamaterial structure loaded with hexagonal pyramid style mass blocks on a square thin film, designed to verify the accuracy of the prediction results of STL of mass blocks with arbitrary shape and area density, are presented.

### 3.1. Cylindrical Mass Block

The film material was polyether amide (PEI) of 20 mm × 20 mm, with Young’s modulus of 2.9 GPa, density of 1270 kg/m^3^ and thickness of 25 μm. The tension was 160 N/m. The cylindrical mass block was a steel column with a mass of 0.2 g and a bottom radius of 2 mm.

**Figure 5:**
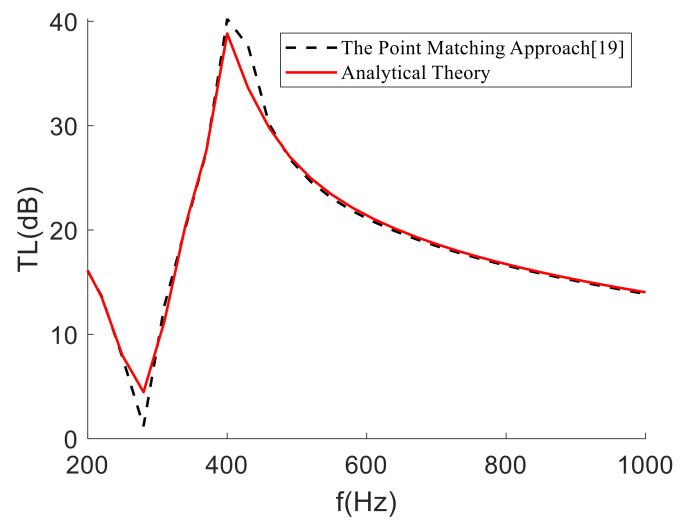
Analytically predicted transmission loss compared with the point-matching method (frequency interval is 30 Hz).

The comparison results are shown in Figure 5. The semi-analytical method in this paper is basically consistent with the theoretical curve in the references. However, the solution in this paper is higher than the literature solution at some frequencies and is smaller than the literature solution at the peak, because the semi-analytical method requires fewer modes than the point-matching method, which leads to higher solutions than those in the literature in some frequency bands; at the same time, the STL effect of the mass block was not considered at the peak, which reduced the result. We used the point matching method and considered the moment of inertia of the mass block to calculate the STL of membrane-type metamaterials. Therefore, a higher number of modes and a more complex linear algebraic method are required, which takes a long time. The semi-analytical method in this paper only needed about 70 s (including the time for calculating the area density of the mass block by finite element software), which has more advantages in engineering.

### 3.2. Hexagonal Pyramid Mass Block

**Figure 6:**
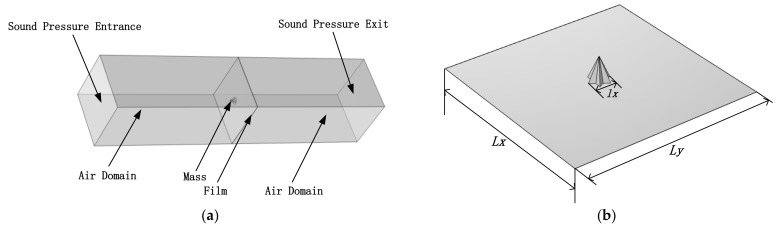
(**a**) System modeled by finite elements for the verification of the analytical mode. (**b**) Hexagonal cone membrane-type acoustic metamaterials.

First, the finite element model was established and the acoustic–solid coupling module in multiphysics field analysis software COMSOL 5.6 was used; the basic modeling model is shown in Figure 6. The model was divided into a square film with a side length (*Lx, Ly*) of 20 mm and a thickness of 25 μm, as well as a membrane-type metamaterial consisting of two hexagonal cones with 2 mm equilateral triangles (*lx*) and a rectangular air domain on both sides of the film. Polyetherimide (PEI) was chosen for the square film material and structural steel for the mass block material, whose parameters are shown in Table 1.

In order to calculate STL, the two sides of the airspace were set as cavities and the length of the cavity was 50 mm, to exclude the influence of the near sound field. The surrounding flanks of the air domain were hard sound field boundaries and the cavity was terminated by a fully absorbing boundary to simulate the situation inside the impedance tube. The membrane was set under a fixed perimeter condition and a prestressed condition of the membrane. Using plane wave radiation conditions, the uniform sound pressure of 1 Pa was perpendicular to the incident surface and the average sound pressure was received on the exit surface to obtain its STL.

In the FE model, the membrane adopted a free quadrilateral mesh, which was swept along the thickness direction; the acoustic cavity was partially based on the free quadrilateral mesh of the membrane, which was further swept; due to its own irregularity, the mass block was obtained by a free quadrilateral mesh. The simulation analysis model of MAM was established physically through the acoustic–structural boundary. The STL of this model is given by Equation (26).

(26) $$TL=20lg\left(P_{in}/P_{out}\right)$$

where $P_{in}$ = 1 Pa and $P_{out}$ is shown in Equation (27).

(27) $$P_{out}=\frac{\int_{s}\left|P_{t}\right|ds}{s}$$

where $P_{t}$ is the total sound pressure of the sound wave exit surface.

**Figure 7:**
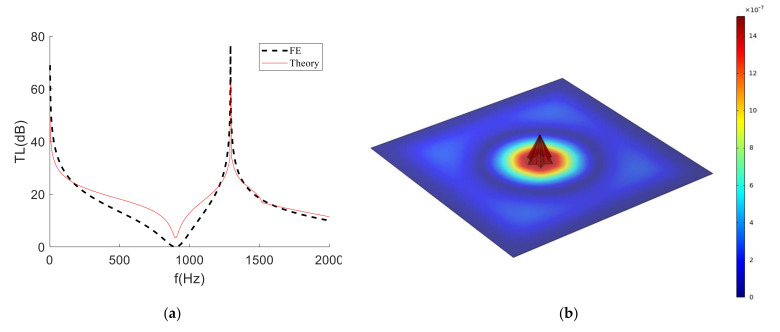
Comparison of simulation and analysis results of hexagonal cone membrane-type metamaterials. (**a**) Comparison of STL between semi-analytical method and finite element simulation. (**b**) Surface vibration velocity of this MAM at 1292 Hz.

As shown in Figure 7, the finite element simulation curve and the theoretical calculation curve had similar trends. In the analysis frequency band, the simulation curve and the theoretical curve were basically the same and the corresponding valley frequency (898 Hz) and the corresponding peak frequency (1292 Hz) could be effectively matched, but the peak and valley values were different. This is because the influence of the stiffness of the mass itself on the film was ignored. In addition, the difference between the two curves was within an acceptable range. Therefore, the established structural theory model could predict the STL effect. It can be seen, from Figure 7b, that, at 1292 Hz, the position with the largest vibration speed was the mass, the surface vibration speed of the film had approached 0 m/s and the maximum vibration speed of the mass did not exceed 10^−7^ m/s. As a result, the sound was well isolated around this frequency.

## 4. Discussion

In this section, the effect of the factors causing different surface density mass blocks on STL is investigated. First, the effect of the longitudinal section inclination on STL is explored. After that, the effect of hexagonal cone with different defects is investigated and the causes of this effect are briefly discussed.

### 4.1. Longitudinal Inclination Angle

**Figure 8:**
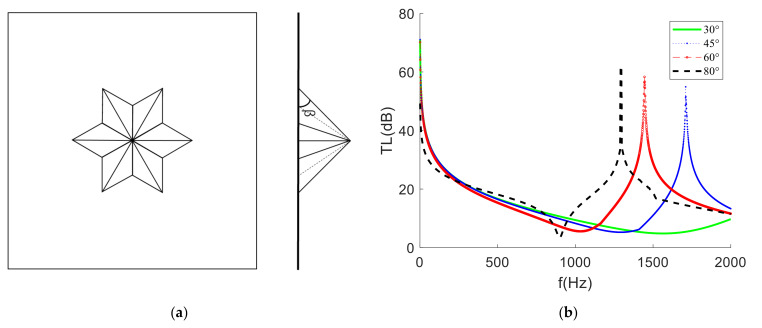
(**a**) The middle section of the membrane-type metamaterial. (**b**) The STL curve at different pitch angles.

Taking the cone-shaped mass block as an example and changing the longitudinal inclination angle of the mass block, it was found that the frequency and size of the STL peak were shifted, as shown in Figure 8. The greater the angle of inclination was, the more concentrated the mass in the center, the more obvious the valley of the sound insulation peak and the closer it was to the peak, with the overall peak and valley shifting towards low frequencies; the smaller the inclination angle was, the more even the mass distribution of the mass block, with the extreme value of STL moving to the high frequency and the change trend also being more gentle (when the inclination angle was 30°, the sound insulation peak shifted out of the analysis frequency band). In other words, the more the areal density was concentrated in the middle of the structure, the greater the peak sound insulation and the lower the frequency of occurrence.

### 4.2. Different Defect States of Hexagonal Cone

**Figure 9:**
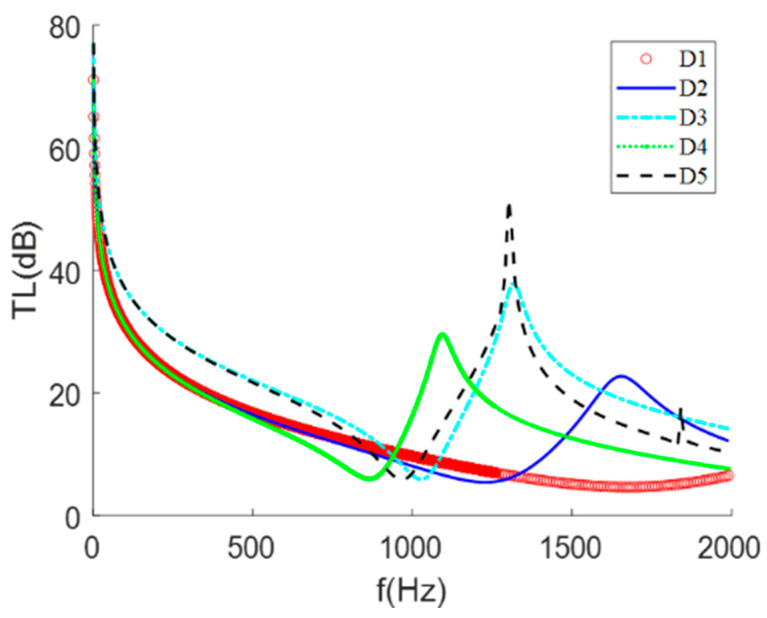
Acoustic transmission loss of each defect state structure.

The MAM loaded with the hexagonal cone mass is called the complete state. The defect states of this membrane-type metamaterial are constructed by dividing the loaded hexagonal cone mass block into six identical rhombic cones in the form of missing *n* (1 ≤ *n* ≤ 5) rhombic cones; STL is shown in Figure 9.

It can be seen from this that, except for the defect state 1, which did not stimulate the sound insulation effect of the structure in the low-frequency band due to low quality, the other defect states all showed a sound insulation effect different from that of the complete hexagonal cone; the sound insulation capacity was lesser than that in the complete state. However, as the defect state structure approached to the complete state, its acoustic-transmission-loss curve gradually approached the complete state.

**Figure 10:**
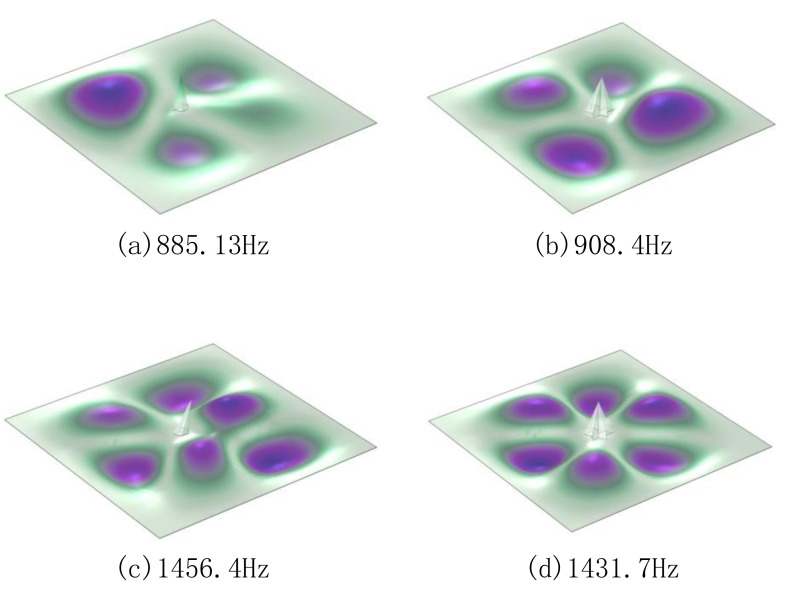
Modes of some membrane-type metamaterials: (**a**) the modal of D2, which existed near the STL curve valley of D5; (**b**) the modal of D5, which existed at the STL curve valley; (**c**) the modal of D2, which was near the STL peak value of D5; (**d**) the modal of D5, which existed in the mode of STL peak.

To analyze the reasons for these results, the characteristic modes of membrane-type metamaterials with different defective structures were studied. The partial modal results of some defective structures are shown in Figure 10.

Taking the modes of defect state 2 and defect state 5 as examples (see Figure 10), it is obvious from the results that the STL curve was affected by different masses. At around 900 Hz, the average displacement of the film in defect state 5 was not zero, compared to defect state 2, so a large amount of sound power could be transmitted and STL valleys were generated. At the peak of the sound-transmission-loss curve of defect state 5, its mode was symmetrical and the amplitude values were similar, so that the average displacement was about 0; therefore, a peak appeared on the STL curve and the defect state 2 had an obvious displacement of the film in a certain position in the mode near this frequency.

In addition to mass, an important reason for these modal results was the difference in the loading position of the mass block. As a result, a coordinate system was established with the center of mass of the film as the origin, the midpoint of the film edge length as the x- and y-axes and the direction of the loading mass block as the positive direction of the z-axis (see Table 2).

The relative position (distance, angle) of the mass block and the film’s center of mass affected STL. When there was a certain linear distance between the mass center and the film’s center of mass, a second obvious STL peak appeared.

**Figure 11:**
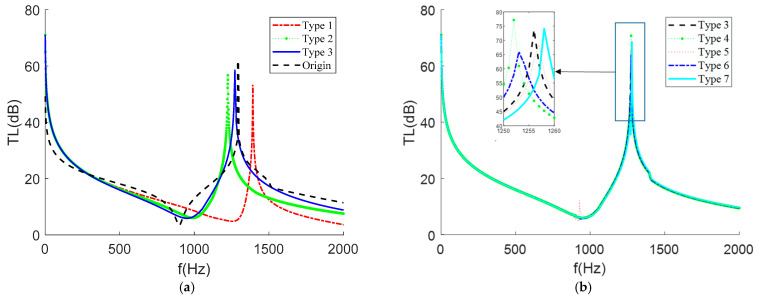
The STL when the masses were distributed in different positions of the film. (**a**) STL when the mass was distributed along the x-axis from the center of mass of the film. (**b**) Since the square film is a symmetrical figure, only the STL when the angle between the center of mass of the film and the mass was rotated from 0 to 45° was studied.

In order to explore the influence of mass eccentricity on STL, seven thin-film metamaterial structures (with the same hexagonal cone mass as the theoretical verification) with different distances and angles from the centroid of the film were designed, as shown in Table 3, and the calculation results are shown in Figure 11.

It is easy to see from Figure 11a that, when the mass block was placed eccentrically, its STL curve was shifted, but the magnitude of the main peaks and valleys (the peaks and valleys produced when the mass is coincident with the film center of mass) and the frequency bands generated did not change much; when the mass block was placed too close to the film boundary, its STL was significantly shifted. Whereas this differs from the behavior of the cylindrical mass block, which produces more peaks and valleys when eccentric [7,8,9,12,13,14,15,16,17,18,19,20], these additional sound insulation summits affected the frequency band and size of the main peaks. It was shown that the hexagonal cone mass block had a more stable sound insulation effect in the lower frequency band. This was also evidenced by the trend in the STL curves for Types 4–7, where changing the mass block angle only resulted in a shift of a few Hz in the peak. This shows that the STL of the membrane-type metamaterials loaded on a single mass block were mainly influenced by the distance between the two mass centers and the relative boundary distance and that they were largely independent of the angle between the mass centers of the two objects.

### 4.3. Films of Different Sizes

**Figure 12:**
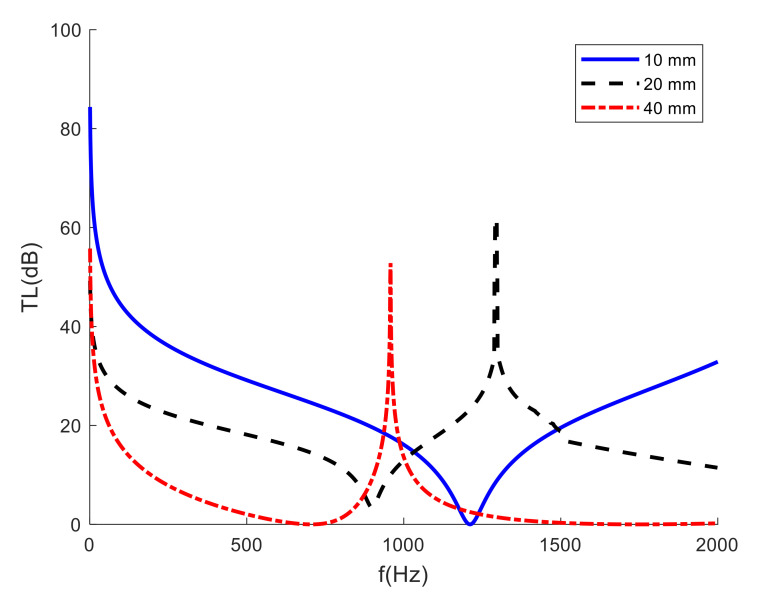
STL curves for different film sizes.

In addition to the influence of the mass on the STL of MAMs, the size of the film is also closely related to its sound transmission loss. In order to explore the effect of film size, three MAM models with side lengths of 10 mm, 20 mm and 40 mm were established and the STL results are shown in Figure 12.

It can be found from Figure 12 that, with the expansion of the film side length, although the trend of the STL curve was similar, the MAM peak gradually moved to the low frequency (when the side length was 10 mm, the sound insulation peak obviously appeared outside the analysis frequency band); when the length was doubled, in some frequency bands, such as 200–400 Hz, the sound insulation value decreased by nearly double. Therefore, when designing the structure of the MAM, it is necessary to select the appropriate film size according to the required sound insulation frequency band and sound insulation size, in order to achieve a better sound insulation effect.

## 5. Conclusions

In this paper, a semi-analytical model is proposed to calculate a membrane-type metamaterial loaded with arbitrarily shaped surface density mass blocks; the model solves the STL prediction of arbitrarily shaped surface density mass blocks while ensuring computational efficiency. Unlike Langfeldt [18], who needed to calculate the effect of mass blocks on the film, the use of conformal mapping theory to map irregular mass blocks into regular mass blocks simplifies the computational difficulty of the theory. After mapping mass blocks of arbitrarily shaped surface density at different locations to the center of the film, the Galerkin method of reference [16] was used to achieve fast prediction of acoustic transmission loss in the low-frequency band of membrane-type metamaterials at vertical incidence of sound pressure. In order to verify the accuracy of the theory, a loaded hexagonal cone-shaped membrane-type material was constructed and the correctness of the analytical solution was verified by relying on finite element simulation results.

In addition, the effect of the change in mass block surface density on STL was investigated and the change in STL when the mass block was located in different positions on the film was analyzed.

(1) The more the areal density was concentrated in the middle of the mass block, the greater the peak sound insulation and the lower the frequency of occurrence.
(2) The quality of the mass block affected the size of the sound insulation peak and the position of the center of mass affected the number of peaks of the curve.
(3) The STL of a single mass block membrane-type metamaterial was mainly influenced by the distance between the mass block center of mass and the film center of mass, as well as the distance between the mass block relative to the film boundary, and was basically independent of the angle between the two objects’ centers of mass.
(4) When the mass of the proof mass was constant, the film size controlled the magnitude of STL and the frequency of the peaks.

In order to realize the industrial application of MAMs, forming large-scale structures by periodically arranging the small-sized unit cells as discussed in this paper should be considered. Ref. [19] showed that the STL theoretical predictions of MAMs are essentially extrapolable to larger constructs consisting of multiple MAMs. At the same time, the overall shape of the large-scale structure and different boundary conditions can have a certain negative impact on the STL of MAMs. In addition, the effects of oblique incident sound field and diffuse incident sound field cannot be ignored. Therefore, considering the conformal matching and boundary conditions of the structure is the key to the further exploration of the STL theory of membrane-type acoustic metamaterials. To better describe the method of transforming a mass of arbitrary shape areal density into a regular mass, the model proposed in this paper only considers the normal incidence of plane waves.

## Figures and Tables

**Table 1:** Material parameter table.

Material	Young’s Modulus (E)	Poisson’s Ratio (nu)	Density (ρ)
PEI	2.9 GPa	0.44	1270 kg/m^3^
Structural steel	210 GPa	0.30	7860 kg/m^3^

**Table 2:** The centroid coordinates of each defect state structure.

Defect State	1	2	3	4	5
x (mm)	0	0.188	−0.25	0.188	0.075
y (mm)	0.433	0.325	0.144	0	0.043
z (mm)	0.075	0.075	0.075	0.075	0.075

**Table 3:** Seven film metamaterials with different mass distribution parameters: Types 1–3 explore the influence of the distance between the centroids; Types 3–7 explore the possible influence of the different included angles between the two centroids.

Type	1	2	3	4	5	6	7
Angle (°)	0	0	0	10	20	30	45
Distance (mm)	7.5	5.0	2.5	2.5	2.5	2.5	2.5

## Data Availability

The data used to support the findings of this study are included within the article
